# Oncology nurses’ compassion fatigue, burn out and compassion satisfaction

**DOI:** 10.1186/s12991-020-00272-9

**Published:** 2020-03-31

**Authors:** Reem Ahmad Jarrad, Sawsan Hammad

**Affiliations:** 1 Clinical Nursing Department, School of Nursing, The University of Jordan, Amman, 11942 Jordan; 2 Community Department, School of Nursing, The University of Jordan, Amman, Jordan

**Keywords:** Oncology nurses, Compassion fatigue, Burnout, Compassion satisfaction, Leisure

## Abstract

**Background:**

For oncology nurses, compassion fatigue, burn out and compassion satisfactions are frequently experienced psychosocial consequences of the oncology work environment. Surveying such phenomena helps to understand how nurses feel and behave when cancer care is provided. Besides, tracking the evolving nature of those three concepts can lend a hand for the early detection of personal and professional suffering of nurses while offering some healing remedies to their struggling bodies and souls.

**Purpose:**

The purpose of this study was to explore the level of compassion fatigue, burn out and compassion satisfaction among a group of specialized oncology nurses. Besides, this study aimed to detect some probable interesting inferences between compassion satisfaction and the concept of rest and leisure. Correlations between compassion fatigue, burn out and compassion satisfaction were investigated. Correlations between oncology nurses’ scores on the three subscales and a group of demographic, organizational and leisure-related variables were examined.

**Methods:**

This study adopted a descriptive correlation design to survey compassion fatigue, burn out and compassion satisfaction among a convenient sample of 100 oncology nurses who work in a specialized cancer care centre. Participants completed compassion fatigue self-test developed by Figely (Compassion fatigue, New York: Brunner/Mazel. B. HudnallStamm, Traumatic Stress Research Group; 1995–1998. http://www.dartmouth.edu/~bhstamm/index.htm, 1995) and a literature-based demographic survey. Analysis of data included descriptive statistics and Pearson correlation co-efficient.

**Results:**

Nurses reported a low level of compassion satisfaction, moderate risk for burn out and an extremely high risk for compassion fatigue. Results revealed significant negative relationships among compassion satisfaction and the number of dependents per nurse. Additionally the correlation between compassion satisfaction and the nurses’ number of hours slept was positive. Only two components of the concept rest and leisure yielded statistical significance when correlated to the concept of compassion satisfaction. A significant negative relationship was observed between compassion satisfaction and compassion fatigue while a strong positive relationship was observed between compassion fatigue and burn out.

**Conclusions:**

The studied oncology nurses sample had evidently low level of compassion satisfaction when contrasted to the significantly increased risks of burn out and compassion fatigue. Thus, health authorities and management are advised to care, in a holistic approach, for nurses who work in oncology departments. Staff-oriented services that offer comfort, reward, leisure, screening, consultation and support are urgently recommended.

## Background

Oncology nurses are encountering a considerable magnitude of day-to-day workplace incapacitating stressors, demands and challenges. These challenges and stressors can be, at least partially attributed to the perception that some of the clients being managed for cancer have terminal illnesses and may die soon [1]. Therefore, oncology centres should put forth more effort to manage the psychosocial consequences of the work environment in an attempt to retain oncology nurses and progress their professional quality of life as well as their overall wellbeing [2].

Compassion fatigue (CF) is a work-related psychosocial consequence which may occur as a result of exposure to a cumulative level of trauma and is emotionally induced by dealing with those who have been traumatized [3]. Another work-related negative consequence is burnout (BO). BO can happen alone or in combination with CF and is a cumulative process marked by emotional exhaustion associated with workload-related stress [4]. A different, though related concept is compassion satisfaction (CS) which is the pleasant feeling derived from lending a hand to others through life’s traumatic situations [5].

Hooper et al. [6] found that oncology nurses were more vulnerable to CF than nurses working in emergency departments. Potter et al. [7] found that the risk of CF was relatively equal between inpatient and outpatient oncology nurses. Evidence suggests that fresh career oncology nurses endure communicating with clients and families, however long hospitalizations and high death rates can provoke CF. The classical picture of CF includes living with clients’ or family members’ emotional pain and fear, getting overwhelmed by a touching client death, and feeling consumed with the responsibility of caring for oncology clients who have endless continuum of physical and psychological needs [8].

BO is a syndrome of emotional exhaustion and reduced personal value [9]. Nurses moving through the torturing experience of BO show psychosomatic, emotional and behavioural cascades. Examples of such problems include, but not limited to weakness, insomnia, anxiety, depression, hostility, apathy, distrust, aggressiveness, irritability and isolation [10].

Elements of BO, particularly emotional exhaustion, were found to be related to the time spent working in the health care field [11]. First, it is noted that nurses with short field experience want to stand out quickly [12]. In reality this is unlikely; therefore new nurses become disappointed and emotionally burn out much faster than experienced nurses [13]. Second, having dependents to care for had a negative impact on oncology nurses experiencing emotional exhaustion as reported by Akyüz [14] and Kutluturkan et al. [13].

Compassion satisfaction (CS) is gaining sense of achievement with content from caring while resisting the negative emotional effects faced in highly complex work environments, such as cancer care agencies [15]. Despite the difficulties, professional oncology nurses can sustain a considerable magnitude of CS by establishing “meaningful memories” (experiences which can help them understand and stand life occurrences in a more courageous and wise way) [16]. Evidence indicates that it is probable for some care givers to maintain caring powers regardless of peaking work pressure and exposure to traumatic situations [17].

Walker and Avant [18] stated that CS-associated feelings include fulfilment, reward, achievement, happiness, enrichment, inspiration, energy, gratitude and hope. While Hinderer et al. [19] found the following elements to have statistically significant relationships with the degree of CS, which are: use of professional counselling, co-worker dynamics, time spent in direct client care, longer duration in current position, increased work hours per shift, older age, decreased level of education, hobbies, quality of social supports, exercise and medications’ usage.

There is insufficient local literature exploring BO, CF and CS among nurses specially those working in oncology care. Besides, attempting to connect the concept of rest and leisure to the positive concept of CS of an oncology caregiver is a very unique approach to explain the differences in scores. Providing such baselines will definitely encourage enthusiastic nursing and health managers, leaders and policy-makers to design future prevention and rescue plans which can help oncology nurses into obtaining higher standards of work environments and better professional quality of life with less work-related negative consequences.

### Theoretical framework

Leisure is defined as the time when you are not working or doing other duties. While leisure activities are defined as the activities you may do while not working. For example; swimming, going to picnics to the forest or the mountains or the sea shore, listening to concerts, going to cinema, travelling abroad, sleeping, surfing the internet, etc. [20].

Recently, happiness studies are becoming more supportive for the idea that leisure enhances subjective wellbeing and its components such as satisfaction [21]. The related theoretical framework supposes that “leisure structural activity” encompasses time spent outside obligated work time; and the typical “leisure activities” include time spent during non-work, while the “subjective engagement of leisure” is subjectively defined as leisure participation and leisure frequency [22].

There is a strong theoretical intersection between the concept of CS and the concepts of happiness and wellbeing which are hypothetically enhanced by practising *less work activities* and *more non*-*work ones* [23]. One major assumption in this research work assumes that if oncology nurses practise less work activities, and more leisure activities; or at least if they have enough chances to practise rest and leisure activities, this will probably help them transcend into a higher wellbeing, happiness and CS states.

### Study aims

This study was designed to explore the levels of CF, BO and CS among a group of specialized cancer care centre oncology nurses. The specific aims were to: (1) describe the level of CF, BO and CS among specialized group of oncology nurses; (2) determine if there were correlations between the oncology nurses’ scores on the three subscales describing their CF, BO and CS levels; (3) identify if there were correlations between oncology nurses’ scores on the three subscales based on some categorized clusters of demographic, organizational and leisure-related variables, namely: age, number of dependents, work hours, sleep hours, days off, eating behaviour as reflected by appetite strength, vacation activities (e.g. travelling during holidays), hobbies and transportation availability to work place.

### Hypotheses

The first hypothesis assumed that oncology nurses have a low level of CS and high levels of CF and BO. Second hypothesis assumed a negative correlation between CS and CF and a positive correlation between CF and BO. The third hypothesis assumed that less rest and leisure activities (i.e. non-work activities) practised by oncology nurses (for example sleep, vacation, hobbies…) may be correlated with lower CS scores.

## Methods

### Design

This study adopted a descriptive correlation design to survey CF, BO and CS among a group of oncology nurses who work in a specialized cancer centre.

### Measures

The participants filled a personal survey which asked questions regarding age, duration of experience, gender, unit, marital status, number of dependents and income. The other group of questions described the nurses’ rest and leisure status as represented by some selected variables. These variables were number of daily work and sleep hours, days off, vacation activities, appetite strength, practising regular hobbies and transportation availability (work commuting). The personal survey was based on literature and required 10 to 15 min to be completed along with a preceding consent form signing, illustrative hand out, a full explanation of the research purpose, methods, and participation rights like confidentiality, right to withdraw, anonymity and privacy.

### Instrument

The CS, BO and CF scores were surveyed using a validated Arabic version of the 66-item CF instrument (Additional file 1: Appendix S1) [24]. This instrument has been used by many researchers with groups of nurses, and showed a strong alpha value (.87–.9) [25, 26].

Each one of the 66 items is evaluated by the care provider on a six-point Likert scale, graded from zero to five. The CF score is the sum of 23 items. The compassion satisfaction score is the sum of 26 items, while the BO is the sum of 16 items [24, 26]. Permissions to use the instrument (the Arabic translated and the original English version) were officially obtained as appropriate.

### Sample and data collection

Convenience sampling was used to recruit 100 nurses from a specialized Cancer Care Centre. The participants were nurses from 8 different units (Table 1). The sampling process included a pilot of 10 nurses which was excluded from the analysis and used only to check comprehensiveness and easiness of the surveys (CF self-test and the personal data sheet). Very minimal language issues were raised and taken care of.

**Table 1:** Distribution of nurses from different units (*n* = 100)

Unit	Acute care units	Bone marrow transplant	ER	Medical wards	Surgical wards	Palliative care	VIP	Paediatric ward
Nurses	12	19	8	19	27	8	3	4

Inclusion criteria included being a registered nurse working in the current unit or floor for more than 3 months at the time of data collection. Questionnaires, illustrative hand outs and consent forms were distributed during nurses’ free times by a well-trained and highly experienced research assistant who holds a master degree in psychiatric nursing. The distributed instrument was in Arabic language. The response rate was 50%, so 200 surveys were distributed, 100 surveys were collected in a 4-month duration. No incentives were given to nurses who took part in the study. The low response rates were explained by the research assistant stating that the nurses were not willing to spend that time in filling papers.

### Setting

The setting from where the data was collected is considered one of the pioneering cancer centres in the Middle East. The centre is located in the capital city Amman, in Jordan. It was established as a national, nongovernmental, independent and non-profit health organization. It receives more than 3500 new cancer care cases and performs 100 bone marrow transplants every year. It has 18 acute care units and variable outpatient clinics which receive 140,000 visits yearly. Services extend from early detection, diagnosis, treatment to palliative care and pain management. Their staff includes 240 oncologists, 2500 technicians, managerial and care specialists and more than 600 nurses. The centre has received many prestigious awards and is working toward Magnet accreditation.

### Statistical analysis and results

#### Sample

The sample consisted of 49 males and 51 females. Their mean age was 25.7 years (SD = 3.8). Majority of the participants (67) were not married and had income equal to 600 JD or less. They had a mean of 3.8 years of experience as nurses and 2.3 years of experience in the current unit/ward. Number of dependents financially supported by nurses ranged from 0 to 8 (Table 2).

**Table 2:** Description of personal demographics, *n* = 100

Variable	*n* (%)	*M* (SD)
Gender		
Male	49 (49)	
Female	51 (51)	
Age		25.7 (3.8)
Experience as a nurse		3.8 (2.3)
Experience in the current unit/ward		2.3 (1.9)
Marital status		
Married	33 (33)	
Not married	67 (67)	
Income		
Less or equal 600	67 (67)	
More than 600	33 (33)	

#### Rest and leisure

The nurses’ daily working hours versus daily sleeping hours had a mean of 11.3 (SD = 1.7), 6.5 (SD = 1.4), respectively. 46% of nurses had enough days off, good appetite (44%), spent outdoors times (36%) or had hobbies (15%). Transportation was considered easy by 55% of the participants (Table 3).

**Table 3:** Description of rest and leisure, *n* = 100

Variable	*n* (%)	*M* (SD)
Rest and leisure		
Daily work hours		11.3 (1.7)
Daily sleeping hours		6.5 (1.4)
Enough days off		
Yes	46 (46)	
No	54 (54)	
Good appetite		
Yes	44 (44)	
No	56 (56)	
Vacations		
Yes	36 (36)	
No	64 (64)	
Hobbies		
Yes	15 (15)	
No	85 (85)	
Availability of transportation to work		
Yes	55 (55)	
No	45 (45)	

Table 4 demonstrates the level of CS, BO and CF among oncology nurses. The nurses reported a low level of CS with a mean of 71.8 (SD = 16.0), moderate risk for BO with a mean of 39.5 (SD = 11.0) and an extremely high risk for CF with a mean of 50.8 (SD = 16.9). These findings are in alignment with the first stated research hypothesis (Table 4).

**Table 4:** Compassion satisfaction, burnout and compassion fatigue among nurses, *n* = 100

Variable	*M* (SD)	Minimum–maximum
Compassion satisfaction	71.8 (16.0)	29–115
Burnout	39.5 (11.0)	15–75
Compassion fatigue	50.8 (16.9)	17–102

#### Correlations among study variables

Table 5 demonstrates the Pearson correlation among the participant’s age, sleeping hours, work hours, dependents, days off, hobbies, appetite, vacation, transportation, CS, BO and CF. The results revealed statistically significant relationship among CS and both: number of dependents (*r* = − .230, *P* = .012) and number of nurses’ sleeping hours (*r* = .212, *P* = .036). A statistically significant negative relationship was observed between CS and CF (*r* = .294, *P* = .003) while a strong positive relationship was observed between CF and BO (*r* = .688, *P* < .001). There was no other significance discovered in the remaining study variables (Table 5).

**Table 5:** Correlations among study variables

Variable	CS *r* (*P*)	CF *r* (*P*)	BO *r* (*P*)
Age	.008 (.93)	− .06 (.55)	− .033 (.75)
Number of dependents	− .230* (.021)	.101 (.31)	.061 (.54)
Work hours	.101 (.3)	.039 (.69)	− .025 (.8)
Sleep hours	.212* (.036)	.120 (.24)	− .025 (.8)
Days off	.007 (.942)	.172 (.087)	.228* (.23)
Appetite	− .186 (.064)	.179* (.49)	.009 (.92)
Vacations	− .056 (.58)	.43 (.67)	.066 (.52)
Hobbies	.156 (.121)	− .92 (.36)	.044 (.66)
Transportation	.061 (.55)	.110 (.28)	.008 (.94)
BO	− .244 (.014)	.688*** (.000)	
CF	− .294** (.003)		

*CS* compassion satisfaction, *CF* compassion fatigue, *BO* burn out

Significant result at: * *P *< .05, ** *P *< .01, *** *P *< .001

## Discussion

### Levels of CF, BO and CS

Oncology nurses in this study reported a low level of CS, moderate risk for BO and an extremely high risk for CF. Jones et al. [27] indicate that the oncology working environment is often stressful and overwhelmingly negative. Oncology nurses experience that kind of workplace negativity, along with a heavy load of job demands and countless number of inevitable effecters as dealing with the dying process and the surrounding grief, tears and suffering, and communicating, all the time as well as in the most critical times, with clients and families. Thus, oncology nurses are less likely to be compassionately satisfied as well as more likely to suffer from CF and BO [28].

Jang et al. [29] state that oncology nurses are more likely to suffer from job-related alcoholism, depression, drug abuse, suicide, CF and BO. Some of the predictable examples of factors that may play a role in manifesting such problems and more are: personnel shortages, frequent new comers who require monitoring and training, lack of managerial support, inter-personnel conflicts, exhausting accreditation processes, punishment systems, lack of job security and lack of visible personal achievement. Furthermore, there are high patient acuities, suffering and mortalities, hopelessness, lack of authority, family bullying, high sense of scrutiny, high work burdens in the shadows of absence of rewarding environment and lack of compensating and stress-ventilation rest and leisure activities [30–32].

It is noteworthy that most of the study participants were young with less than 5 years of experience in nursing and oncology (this low experience time may be attributed to a possible organizational trend towards hiring fresh graduates, a relatively high turnover rate, and clinic staff were not surveyed). Despite this, the study participants showed signs of CF and BO and decreased CS. It is recommended to study the specific contributing factors to this phenomenon in order to facilitate future prevention and resolution of such alarming oncology care institutional problems.

### Correlation between CF, CS and BO

Statistically significant negative relationships were observed between CS and CF which is consistent with the reports of Slocum-Gori et al. [33], Pelon [34] and Zhang et al. [35], while a strong positive relationship was observed between CF and BO and is consistent with what was found by Sung et al. [36], Slocum-Gori et al. [33], Pelon [34] and Zhang et al. [35]. Both results were consistent with the related initial hypotheses. Consequently, it is reasonable to conclude that, for the nurses who care for cancer clients, lower CS can bring higher CF. Higher CF can drag oncology nurses to the dangerous edges of BO [37].

Such serious correlations make it mandatory for authority figures and oncology nursing policy-makers to create more supportive and welcoming work conditions for those who care for oncology clients. They need to tailor staff-oriented interventions to act as protective mechanisms. For example, staff psychiatric and professional counselling on a regular basis, holistic self-care activities, positive coping skills training, physical exercise, meditation, self-reflection, rest, play therapy, mindfulness-based approaches and relaxation techniques such as yoga, Tai Chi and Reiki [38, 39].

### Correlation among CS and rest and leisure

It was evident in this study that only two elements of the “concept of rest and leisure” had been correlated with the level of CS. These two elements are the number of nurses sleeping hours and number of dependents. These results partially matched our hypothesis which assumed that less rest and leisure activities practised by oncology nurses would be correlated with lower CS scores.

It should be mentioned here that the studies investigating CS-correlating factors are limited in nursing, so further focus from other researchers should be exerted in this area. Literature was relatively supportive for the positive correlation between CS and rest and leisure. For example, having dependents to care for (physically, psychologically and financially) had an impact on oncology nurses feeling emotional exhaustion as reported by Akyüz [14] and Kutluturkan [40]. Many nurses, go through a second exposure to stress in a “2nd daily shift” defined as caregiving at home with responsibility for sons, daughters, spouses and elderly family members, hence decreasing their overall CS level [41].

Sleep is a very crucial concept in this area. Simply, lack of sleep will yield a less compassionately satisfied nurse. The other facet of the same coin is long working hours, followed by long hours at home as the primary homemaker and caregiver. These two factors will definitely result in less sleeping hours [19]. As mentioned in this study, working hours were 11.3 (1.7) when compared to sleeping hours as low as 6.5 (1.4). The National Sleep Foundation [42] generally recommends 7–10 h of sleep per night for adults to maintain a functional health.

Nurses who work at night or in rotating shifts rarely have sufficient amounts of sleep. Sleep loss is cumulative, and after a while, the sleep debt may be significant enough to impair integration of information, cloud decision-making, and interrupt planning, plan implementation and vigilance [43]. Insufficient sleep has been associated with mood and cognition changes, lower quality job performance, declined motivation, safety risks and physiological complains [44, 45]. It is not surprising to know that none of the several studies reviewed, over the past 20 years, suggested *any* positive effect gained from sleep deprivation/insufficiency in healthy adults. To sum up, depression, irritability and less CS in nurses significantly peak when sleep is restricted [46].

### Study limitations

It should be mentioned that there were some limitations in our study. For example, the sample size was relatively small; larger size replication studies are encouraged to better detect correlations and differences. Convenience sampling was adopted which could limit generalizability of the findings to the whole oncology nursing community in Jordan. The participating nurses were from many departments, so the subgroups were very small; this made it difficult to run subgroup comparisons to see if there were differences in CF, CS, BO scores between them.

Future studies are encouraged to run subgroup comparisons, increase sample size, adopt randomization, adopt multi-centre approach, or make regional or international comparisons, and study a wider range of organizational, personal and environmental oncology nursing variables. Such research strategy will surely enhance more in-depth insights. Combining qualitative sections are recommended as well to better understand the phenomena of CF, CS and BO in the area of oncology nursing.

## Conclusions and recommendations

It could be concluded that oncology nurses in Jordan are under a great deal of stress, life and work burdens which leave them defenceless against the winds of CF and BO with less CS. This research paper had identified the presence of a serious problem, accordingly, immediate action plans must be set.

We can make a few suggestions here. For example, to partially improve CS, sleep enhancing practices should be promoted among nurses, such as less caffeine intake, night shifts to be scheduled in a reasonable pattern, cutting night shifts into shorter durations, offering nurses official sleeping naps during night shifts [44], establishment of a supportive climate within the unit, providing physical exercise services and a nice eating healthy facilities within the hospital setting [47]. Children and old age day and night care services can be offered for nurses in manageable fees, in an attempt to minimize family care burdens and increase nurses available rest time.

### Implications for nursing management and policy-makers

Policy-makers in the area of oncology nursing are urgently invited to open a dialogue with nurses and nursing bodies, conduct institutional and national surveys and interviews to identify causative and contributive factors related to CF, CS and BO. This could serve as an attempt to find best practices to *win* better outcomes for nurses, clients and institutions.

A few suggestions in this regard may include: eliminating personnel shortages, assigning “free load staff” to train the new comers [48] and demonstrating evidence of managerial support when needed/expected. Examples of managerial support include: providing special consultation services to help solve inter-personnel conflicts, getting sufficient time and extra staff during accreditation processes, logically revising punishment systems in a way which puts the nurses psychological welfare as a priority, enhancing nurses’ job security through practical and legal procedures along with establishing visible personal achievement, reward and support systems [49]. Moreover, nurses work days could be reduced in number or in length to provide stress-ventilation windows [50]. Such measures may eliminate the risk of developing CF and BO and their well-known personal and organizational negative outcomes.

## Supplementary information

**Additional file 1.** Compassion satisfaction/fatigue self-test for helpers.

## Data Availability

The data sets are available in the primary investigator’s local desk. If any researcher is interested in secondary analysis of data he can email the corresponding author for this purpose.
