# Supplementary material for: Oncology nurses’ compassion fatigue, burn out and compassion satisfaction
Source: Ann Gen Psychiatry. 2020 Mar 31;19:22. doi: 10.1186/s12991-020-00272-9 (PMC7110622; doi:10.1186/s12991-020-00272-9)
Supplement: Supplementary file 1 — Additional file 1. Compassion satisfaction/fatigue self-test for helpers. [file 12991_2020_272_MOESM1_ESM.docx]

**Compassion Satisfaction/Fatigue Self-Test for Helpers**

Adapted with permission from Figley, C.R., (1995). Compassion Fatigue, New York: Brunner/Mazel.© B. Hudnall Stamm, Traumatic Stress Research Group, 1995 -1998 http://www.dartmouth.edu/~bhstamm/index.htm.

*This form may be freely copied as long as (a) authors are credited,(b) no changes are made, & (c) it is not sold.*

Helping others puts you in direct contact with other people's lives. As you probably have experienced, your compassion for those you help has both positive and negative aspects. This self -test helps you estimate your compassion status: This includes your risk of burnout, compassion fatigue and satisfaction with helping others. Consider each of the following characteristics about you and your current situation

| **0**  **Never** | **1**  **Rarely** | **2**  **A Few Times** | **3**  **Somewhat**  **Often** | **4**  **Often** | **5**  **Very Often** |
| --- | --- | --- | --- | --- | --- |

**Items About You**

____1. I am happy.

____2. I find my life satisfying.

____3. I have beliefs that sustain me.

____4. I feel estranged from others.

____5. I find that I learn new things from those I care for.

____6. I force myself to avoid certain thoughts or feelings that remind me of a frightening experience.

____7. I find myself avoiding certain activities or situations because they remind me of a frightening experience.

____8. I have gaps in my memory about frightening events.

____9. I feel connected to others.

____10. I feel calm.

____11. I believe that I have a good balance between my work and my free time.

____12. I have difficulty falling or staying asleep.

____13. I have outburst of anger or irritability with little provocation

____14. I am the person I always wanted to be.

____15. I startle easily.

____16. While working with a victim, I thought about violence against the perpetrator.

____17. I am a sensitive person.

____18. I have flashbacks connected to those I help.

____19. I have good peer support when I need to work through a highly stressful experience.

____20. I have had first-hand experience with traumatic events in my adult life.

____21. I have had first-hand experience with traumatic events in my childhood.

____22. I think that I need to "work through" a traumatic experience in my life.

____23. I think that I need more close friends.

____24. I think that there is no one to talk with about highly stressful experiences.

____25. I have concluded that I work too hard for my own good.

____26. Working with those I help brings me a great deal of satisfaction.

____27. I feel invigorated after working with those I help.

____28. I am frightened of things a person I helped has said or done to me.

____29. I experience troubling dreams similar to those I help.

____30. I have happy thoughts about those I help and how I could help them.

____31. I experienced intrusive thoughts of times with especially difficult people I helped.

____32. I have suddenly and involuntarily recalled a frightening experience while working with a person I helped.

____33. I am preoccupied with more than one person I help.

____34. I am losing sleep over a person I help's traumatic experiences.

____35. I have joyful feelings about how I can help the victims I work with.

____36. I think that I might have been "infected" by the traumatic stress of those I help.

____37. I think that I might be positively "inoculated" by the traumatic stress of those I help.

____38. I remind myself to be less concerned about the well being of those I help.

____39. I have felt trapped by my work as a helper.

____40. I have a sense of hopelessness associated with working with those I help.

____41. I have felt "on edge" about various things and I attribute this to working with certain people I help.

____42. I wish that I could avoid working with some people I help.

____43. Some people I help are particularly enjoyable to work with.

____44. I have been in danger working with people I help.

____45. I feel that some people I help dislike me personally.

**Items About Being a Helper and Your Helping Environment**

____46. I like my work as a helper.

____47. I feel like I have the tools and resources that I need to do my work as a helper.

____48. I have felt weak, tired, run down as a result of my work as helper.

____49. I have felt depressed as a result of my work as a helper.

____50. I have thoughts that I am a "success" as a helper.

____51. I am unsuccessful at separating helping from personal life.

____52. I enjoy my co-workers.

____53. I depend on my co-workers to help me when I need it.

____54. My co-workers can depend on me for help when they need it.

____55. I trust my co-workers.

____56. I feel little compassion toward most of my co-workers

____57. I am pleased with how I am able to keep up with helping technology.

____58. I feel I am working more for the money/prestige than for personal fulfillment.

____59. Although I have to do paperwork that I don't like, I still have time to work with those help.

____60. I find it difficult separating my personal life from my helper life.

____61. I am pleased with how I am able to keep up with helping techniques and protocols.

____62. I have a sense of worthlessness/disillusionment/resentment associated with my role as a helper.

____63. I have thoughts that I am a "failure" as a helper.

____64. I have thoughts that I am not succeeding at achieving my life goals.

____65. I have to deal with bureaucratic, unimportant tasks in my work as a helper.

____66. I plan to be a helper for a long time.
